# Differential Incidence of Tongue Base Cancer in Male and Female HPV16-Transgenic Mice: Role of Female Sex Hormone Receptors

**DOI:** 10.3390/pathogens10101224

**Published:** 2021-09-22

**Authors:** Clariano Pires de Oliveira Neto, Beatriz Medeiros-Fonseca, Diogo Estêvão, Verónica F. Mestre, Natália R. Costa, Fábio Evangelista de Andrade, Paula A. Oliveira, Margarida M. S. M. Bastos, Rui Medeiros, Diogo Assis, Ana Félix, Fernanda Ferreira Lopes, Rui M. Gil da Costa, Haissa O. Brito, Luciane M. O. Brito

**Affiliations:** 1 Post-Graduate Programme in Adult Health (PPGSAD), Federal University of Maranhão, São Luis 65080-805, Brazil; clariano.neto@discente.ufma.br (C.P.d.O.N.); fernanda.ferreira@ufma.br (F.F.L.); haissa.brito@ufma.br (H.O.B.); luciane.brito@ufma.br (L.M.O.B.); 2 Centre for the Research and Technology of Agro-Environmental and Biological Sciences (CITAB), University of Trás-os-Montes and Alto Douro (UTAD), Quinta de Prados, 5000-801 Vila Real, Portugal; al59898@utad.eu (B.M.-F.); al59953@utad.pt (V.F.M.); pamo@utad.pt (P.A.O.); 3 Molecular Oncology and Viral Pathology Group, Research Center of IPO Porto (CI-IPOP)/RISE@CI-IPOP (Health Research Network), Portuguese Oncology Institute of Porto (IPO Porto)/Porto Comprehensive Cancer Center (Porto.CCC), 4200-072 Porto, Portugal; destevao@i3s.up.pt (D.E.); maria.vieira.costa@ipoporto.min-saude.pt (N.R.C.); ruimedei@ipoporto.min-saude.pt (R.M.); 4 FMUP, Faculty of Medicine, University of Porto, 4200-319 Porto, Portugal; 5 Veterinary Sciences Department, State University of Maranhão, São Luis 65081-400, Brazil; fabioandrade@professor.uema.br; 6 Veterinary Sciences Department, University of Trás-os-Montes and Alto Douro, UTAD, Quinta de Prados, 5001-801 Vila Real, Portugal; 7 LEPABE-Laboratory for Process Engineering, Environment, Biotechnology and Energy, Faculty of Engineering, University of Porto, 4200-465 Porto, Portugal; mbastos@fe.up.pt; 8 CEBIMED, Faculty of Health Sciences, Fernando Pessoa University, 4249-004 Porto, Portugal; 9 Research Department of the Portuguese League against Cancer Regional Nucleus of the North (LPCC—NRNorte), Estrada da Circunvalação 6657, 4200-177 Porto, Portugal; 10 Department of Morphology, Federal University of Maranhão, São Luis 65080-805, Brazil; diogo.rubim@ufma.br; 11 Department of Pathology, Instituto Portugues de Oncologia de Lisboa, NOVA Medical School, University NOVA of Lisbon, Campo dos Mártires da Pátria, 130, 1169-056 Lisbon, Portugal; ana.felix@nms.unl.pt; 12 Pathology Department Portuguese Institute of Oncology Lisbon Franscisco Gentil, Rua Professor Lima Bastos, 1099-023 Lisbon, Portugal; 13 UFMA University Hospital (HUUFMA), São Luis 65020-070, Brazil

**Keywords:** estrogen receptor, progesterone receptor, collagen, matrix metalloproteinase, tongue base cancer

## Abstract

A growing proportion of oropharyngeal squamous cell carcinomas (OPSCC) are associated with infection by high-risk human papillomavirus (HPV). For reasons that remain largely unknown, HPV+OPSCC is significantly more common in men than in women. This study aims to determine the incidence of OPSCC in male and female HPV16-transgenic mice and to explore the role of female sex hormone receptors in the sexual predisposition for HPV+ OPSCC. The tongues of 30-weeks-old HPV16-transgenic male (n = 80) and female (n = 90) and matched wild-type male (n = 10) and female (n = 10) FVB/n mice were screened histologically for intraepithelial and invasive lesions in 2017 at the Centre for the Research and Technology of Agro-Environmental and Biological Sciences (CITAB), Portugal. Expression of estrogen receptors alpha (ERα) and beta (ERβ), progesterone receptors (PR) and matrix metalloproteinase 2 (MMP2) was studied immunohistochemically. Collagen remodeling was studied using picrosirius red. Female mice showed robust ERα and ERβ expression in intraepithelial and invasive lesions, which was accompanied by strong MMP2 expression and marked collagen remodeling. Male mice showed minimal ERα, ERβ and MMP2 expression and unaltered collagen patterns. These results confirm the association of HPV16 with tongue base cancer in both sexes. The higher cancer incidence in female versus male mice contrasts with data from OPSCC patients and is associated with enhanced ER expression via MMP2 upregulation.

## 1. Introduction

Infection with high-risk human papillomavirus (HPV) is associated with a number of epithelial cancers, most prominently cervical cancer but also other anogenital cancers and a growing subset of head and neck squamous cell carcinomas, particularly many of those located in the oropharynx (OPSCC) [1]. HPV+OPSCC shows distinctive characteristics in terms of epidemiology, location and biological behavior. For reasons that remain largely unknown, these lesions preferentially affect male patients, with reported male to female ratios varying between 2:1 and 4:1 [2]. The response of HPV+OPSCC to radiation and chemotherapy is generally more favorable than that of HPV-negative lesions with longer survival [3,4,5], and patients with HPV+ lesions may benefit from de-intensified therapeutic approaches with reduced toxicities [6,7,8]. HPV+OPSCC preferentially targets oropharyngeal locations such as the tonsils and the tongue base, and our group recently demonstrated that HPV16 specifically targets the tongue base in transgenic mice [9]. Using mice where the entire early HPV16 genomic region is expressed under the control of the cytokeratin 14 (Krt14) gene promoter, we demonstrated that HPV is sufficient to induce intraepithelial lesions in multiple oral and oropharyngeal sites, but that invasive lesions are restricted to the tongue base. This was associated with the presence of a squamocolumnar junction located in the circumvallate papilla, similar to the anal squamocolumnar junction, which constitutes a transformation zone comprising cells that are uniquely sensitive to HPV-induced transformation [10]. Upon exposure to a chemical carcinogen, squamous cell carcinomas arise at multiple locations, presumably arising from HPV-induced intraepithelial lesions [11]. These studies were conducted in female mice, but male patients are consistently more affected by HPV+OPSCC than women [2]. Previous studies suggested that estrogen receptor alpha (ERα) is associated with HPV presence in OPSCC patients [12], while the opposite is observed for progesterone receptor (PR) [13]. In mice carrying one or more HPV16 transgenes (e.g., HPV16 E6, E7 and/or E5), estrogen signaling via ERα is critical for cervical carcinogenesis [14,15]. Conversely, progesterone signaling via PR blocks cervical carcinogenesis in this mouse model [16]. Therefore, we hypothesized that sex-related differences in OPSCC incidence are associated with different expression levels of female sex hormone receptors, and the present work aims to compare the incidence and distribution of intraepithelial and invasive lesions in male and female HPV16-transgenic mice and to investigate the possible role of sex hormone receptors in determining cancer incidence in both sexes.

## 2. Materials and Methods

### 2.1. Animals

Transgenic mice carrying the entire HPV16 early genomic region under the control of the cytokeratin 14 (Krt14) gene promoter (K14-HPV16 mice) were previously generated on an FVB/n background [12]. This mouse strain was kindly donated by Dr. Jeffrey Arbeit and Dr. Douglas Hanahan, through the USA National Cancer Institute Mouse Repository. The animal experiments were approved by the University of Trás-os-Montes and Alto Douro Ethics Committee (Approval Number 10/2013) and the Portuguese Veterinary Directorate (Approval Number 0421000/000/2014) and carried out at the University of Trás-os-Montes and Alto Douro animal facility. Mice were maintained and bred according to the Portuguese (Decreto-Lei 1005/92 dated 23 October) and European (EU Directive 2010/63/EU) legislation, with 12 h light/12 h dark cycles, at 20–24 °C and 50 ± 10% relative humidity. For reproduction, transgenic males were mated with wild-type females, allowing for conveniently large litters of heterozygous mice. Independent mouse groups were used in two separate experiments for studying the expression of HPV16 oncogenes (qRT-PCR experiments) and for studying the expression of hormone receptors in tongue tissues. Weight loss over 10% in a two week period and/or cutaneous or mucosal ulcers were considered humane endpoints for euthanizing affected mice during the experimental period. 

### 2.2. Genotyping

Tail tip samples were macerated in TRI Reagent (Grisp, Porto, Portugal) and used to determine the presence of the HPV16 transgenes. DNA was extracted and purified using the GRS-Genomic DNA Broadrange Kit (Grisp, Porto, Portugal). DNA concentration and purity were assessed using a NanoDrop v3.7 spectrophotometer (Thermo Scientific, Waltham, MA, USA). The HPV16 E7 and the β-globin (Hbb, used as a loading control) genes were amplified using polymerase chain reaction (PCR) with specific primers (Table S1). 

### 2.3. RNA Extraction and cDNA Synthesis

We first hypothesized that differential cancer incidence could be related to differential expression of viral oncogenes in female and male animals. To test this hypothesis, we conducted an experiment (Experiment 1) in which female (n = 10) and male (n = 10) ten weeks-old K14-HPV16 mice were maintained until reaching 30 weeks-old. The animals were sacrificed using ketamine and xylazine anesthesia, followed by intracardiac puncture and exsanguination. Then, the entire tongue was sectioned sagitally and one half was collected into TripleXtractor (Grisp, Porto, Portugal) for RNA extraction, macerated and kept at −80 °C until further use. The other half was processed for histological analysis to determine the incidence of cancer in male and female animals. RNA was extracted using the High Pure RNA Isolation Kit (Roche, Basel, Switzerland) and treated with DNase I to avoid genomic DNA contamination. First-strand synthesis of mRNA, cDNA, was carried out with the High-Capacity cDNA Reverse Transcriptase Kit (Applied Biosystems, Waltham, MA, USA). All reverse transcriptase reactions included a negative control and were performed in a Mycycler Thermal cycler (Bio-Rad, Hercules, CA, USA).

### 2.4. qRT-PCR 

Real-time PCR was conducted using the Fast SYBR Green Master Mix (Applied Biosystems, Waltham, MA, USA) and performed on a StepOne qPCR Real-Time PCR device (Applied Biosystems, Waltham, MA, USA). The cycling conditions were 95 °C for 20 s, followed by 40 cycles at 95 °C for three seconds and 60–66 °C on the primer annealing temperature for 30 s. An additional 95 °C for 15 s, 60 °C for one minute and 95 °C for 15 s was used to confirm the amplification specificity of each reaction. Additional “no template” controls were used to ensure the absence of genomic DNA contamination. All qPCRs were run in duplicates, and the average standard deviation within all samples and respective duplicates was 0.24. The PCR efficiencies of the mRNA targets were between 94–100%, with a consequent slope variation between 3.28–3.48. HPV16 E6, E7 and E5 mRNA expression levels were normalized to the average expression of two reference genes, TBP and HPRT, using specific primers, as described in Table S2. The expression of TBP, HPRT and β2m genes was analyzed by qRT-PCR in both sexes to choose the most stable reference gene to be used in subsequent tests. The stability value was assessed using NormFinder software, taking in consideration the inter and intra-variability between the samples (β2m = 0.278; TBP = 0.437 and HPRT = 0.477); however, the best combination of endogenous control genes is a combination of TBP and HPRT, with a stability value of 0.221 [17].

### 2.5. Expression of Sex Hormone Receptors: Mouse Experiments

Considering the results of Experiment 1, a larger mouse experiment (Experiment 2) was designed to confirm the distribution of intraepithelial and invasive lesions in HPV16-transgenic male and female mice and study the expression of sex hormone receptors in tongue tissues. Ten weeks-old female (n = 90) and male (n = 80) K14-HPV16 and matched wild-type female (n = 10) and male (n = 10) mice were maintained up to 30 weeks of age. The unusually high numbers of transgenic mice were chosen based on previous studies. Jabbar et al. used similar HPV16-transgenic models and a chemical carcinogen to drive carcinogenesis, with final mouse numbers per group ranging between 20 and 34 [18]. Since the present study does not employ chemical carcinogens but only the HPV16 transgenes, much lower incidence rates were expected, requiring higher animal numbers to achieve meaningful results. 

At the end of the experiment, all mice were sacrificed using ketamine and xylazine anesthesia, followed by intracardiac puncture and exsanguination. Then, the tongue was collected and sectioned sagitally for histological and immunohistochemical examination.

### 2.6. Histology

Samples for histological analysis were fixed in 10% neutral buffered formalin. The fixed tissues were dehydrated through graded alcohols and xylene and embedded in paraffin. Further, 5 μm-thick sections were stained with hematoxylin and eosin for histological classification. Tongue lesions were classified as papillomas, low-grade dysplasia, high-grade dysplasia or invasive squamous cell carcinoma (SCC) based on previously established criteria [9]. Briefly, samples were considered normal if the epithelial architecture was fully preserved. Papillomas were exophytic lesions with prominent acanthosis and basal layer hyperplasia, usually accompanied by marked hyperkeratosis. Low-grade dysplastic lesions showed basal layer hyperplasia with only minor cell atypia and preserved suprabasal differentiation. High-grade dysplasia was diagnosed on the basis of marked cytological atypia with suprabasal mitotic figures and marked anisokaryosis. The diagnosis of SCC was made when groups of epithelial cells invaded the underlying lamina propria of the tongue musculature. Sections stained with picrosirius red were observed under polarized light to assess collagen remodeling in the tongue base area of each sample.

### 2.7. Immunohistochemistry

Twenty mouse samples were selected for IHC (5 female HPV+, five female HPV−, five male HPV+ and five male HPV−). Paraffin-embedded tissue sections were obtained at 5 μm, deparaffinized in xylene, rehydrated in graded ethanol and submitted to antigen recovery using Trilogy solution (Cell Marque) in a pressure cooker for 15 min. Endogenous peroxidase was blocked with 3% hydrogen peroxide. The sections were then incubated with the primary antibody (Table S3), and staining was detected using a commercial (REVEAL, Spring Biosciences, Pleasanton, CA, USA). The immunostaining in the tongue base area of each sample was scored blindly in tumor cells showing nuclear staining. Scores were obtained by multiplying a factor related to stain intensity (0—null, 1—fain, 2—moderate, 3—intense) by the percentage of stained cells (ranging between 0 and 100). The final score ranged between 0 and 300 and was expressed as arbitrary units (AU). For statistical analysis, the scores of low- and high-grade dysplasia were grouped together as intraepithelial lesions.

### 2.8. Statistical Analysis

Statistical analysis was performed using GraphPad Prism 8.4.3. Normality was verified using the Kolmogorov-Smirnov test. Kruskal-Wallis and Mann-Whitney tests were used to evaluate statistical differences in normalized relative expression (−ΔCt) of the HPV16 E6, E7 and E5 genes among the different groups. A Chi-square test was used to study the distribution of the various histological lesions. Mann-Whitney’s test was applied to study the distribution of immunohistochemical scores. Dunn´s test was used to adjust for multiple comparisons. Statistical significance was set at *p* ˂ 0.05.

## 3. Results

### 3.1. Expression of HPV16 E5, E6 and E7 in Male and Female Mice

#### Experiment 1

**Figure 1:**
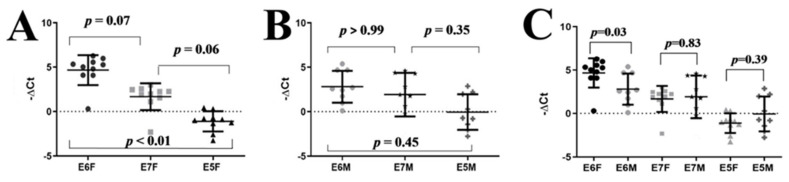
Normalized relative expression (−ΔCt) of HPV 16 E6, E7 and E5 in tongue base samples from K14-HPV16 mice. Panel (**A**) shows data from female mice, Panel (**B**) shows data from male mice, and Panel (**C**) compares oncogene expression in male and female mice. F—female, M—male.

We started by examining the incidence of lesions in small (n = 10) groups of male and female HPV-16-transgenic or wild-type mice on Experiment 1. Wild-type mice showed no lesions. Low-grade dysplastic lesions showed increased cellularity, hyperkeratosis and dyskeratosis, while high-grade lesions also displayed suprabasal mitotic figures, anisokaryosis and severe disruption of the epithelial architecture. SCC invaded the underlying connective and muscular tissues and elicited marked stromal remodeling. Interestingly, the incidence of SCC was slightly higher in HPV16-transgenic female compared with male mice: six female mice showed low-grade dysplasia, two showed high-grade dysplasia, and two showed SCC; in the male group, six mice showed low-grade dysplasia, two showed high-grade dysplasia, and a single animal was diagnosed with SCC. We then compared the mRNA expression of the HPV16 E6, E7 and E5 oncogenes in male and female K14-HPV16 tongue tissue samples. We observed expression of all three oncogenes in tongue tissues. Interestingly, the expression profile of the three oncogenes showed a trend towards a homogenous cascade: E6 mRNA was most abundant, followed by E7 and E5 (E6 > E7 > E5) in male and female mice (Figure 1A,B). The difference between E6 and E5 levels reached statistical significance in female animals (*p* ˂ 0.01). Importantly, E6 mRNA expression was approximately seven-fold higher in females than in males (*p* = 0.03), while E7 and E5 mRNA expression were similar in males and females (*p* = 0.83 and *p* = 0.39 respectively) (Figure 1C). Next, we compared the expression of HPV16 E6, E7 and E5 mRNAs in low-grade lesions versus the more advanced high-grade/SCC lesions, pooling together animals with SCC and high-grade dysplasia. The E6, E7 and E5 mRNA expression levels were similar in both types of lesions in females (*p* = 0.70; *p* = 0.70 and *p* = 0.33 respectively) and in males K14-HPV16 (*p* = 0.90; *p* = 0.62 and *p* = 0.55 respectively) (Figure S1). These results suggested increased HPV16 E6 expression and higher incidence of SCC in females, in contrast with clinical observations from OPSCC patients, and prompted us to confirm lesion incidence in male and female HPV16-transgenic mice, using larger experimental groups, in Experiment 2.

### 3.2. Differential Incidence of Tongue Base Cancer in Male and Female Mice

#### Experiment 2

**Figure 2:**
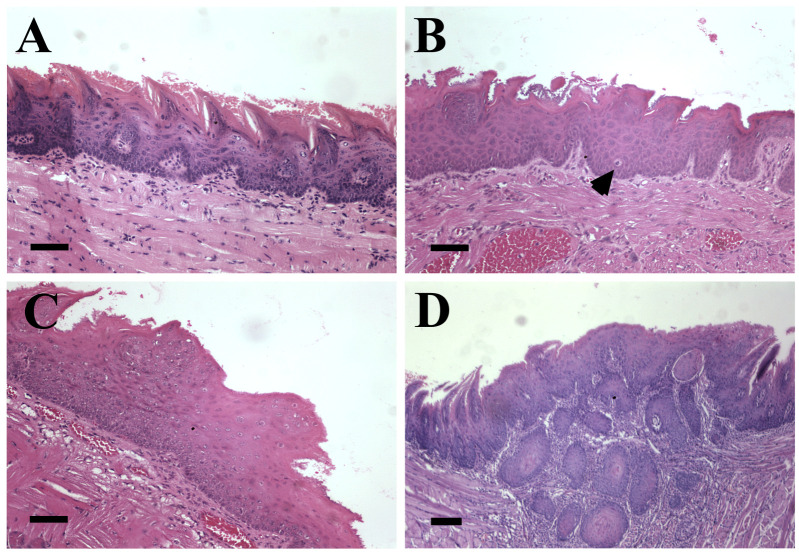
Representative histological images of tongue lesions in K14-HPV16 mice, hematoxylin and eosin (H&E). (**A**) Low-grade dysplasia. Note: increased cellularity of the basal layer, with sustained differentiation of suprabasal layers and minimal anysokaryosis (100×, bar = 100 μm), (**B**) high-grade dysplasia. Note: loss of differentiation of suprabasal layers with anysokaryosis and suprabasal mitotic figures (arrow) (100×, bar = 100 μm), (**C**) papilloma. Note: exophytic lesion with nuclear crowding (100×, bar = 100 μm). (**D**) Squamous cell carcinoma. Note: islands of tumor cells invading the lamina propria and underlying skeletal muscle (40×, bar = 200 μm).

During Experiment 2, no mice had to be sacrificed due to animal welfare concerns. Eight female and 13 male HPV16-transgenic mice died during the experimental period. Necropsy findings were inconclusive concerning the causes of death due to quick and extensive autolytic phenomena. The final numbers of mice in each experimental group are summarized in Table 1. Upon histological examination, a number of lesions were identified in the tongue of female and male HPV16-transgenic mice, which are summarized in Table 1 and Figure 2. Interestingly, the incidence of intraepithelial lesions was similar between HPV16-transgenic males and females, but SCC was approximately five-fold more frequent in female mice (19 of 82 mice) than in matched male (3 of 67) animals (*p* = 0.0071 in a Chi-square test). Concerning the distribution of lesions, papillomas and SCC were mainly located in the tongue base in both male and female mice (Table 2). Conversely, intraepithelial lesions were distributed throughout the tongue. In SCCs, nests of neoplastic cells invaded the underlying lamina propria and skeletal muscle. Neoplastic cell showed moderate nuclear pleomorphism and scarce multifocal keratinization. 

### 3.3. Expression of Female Sex Hormone Receptors in Male and Female Mice

**Figure 3:**
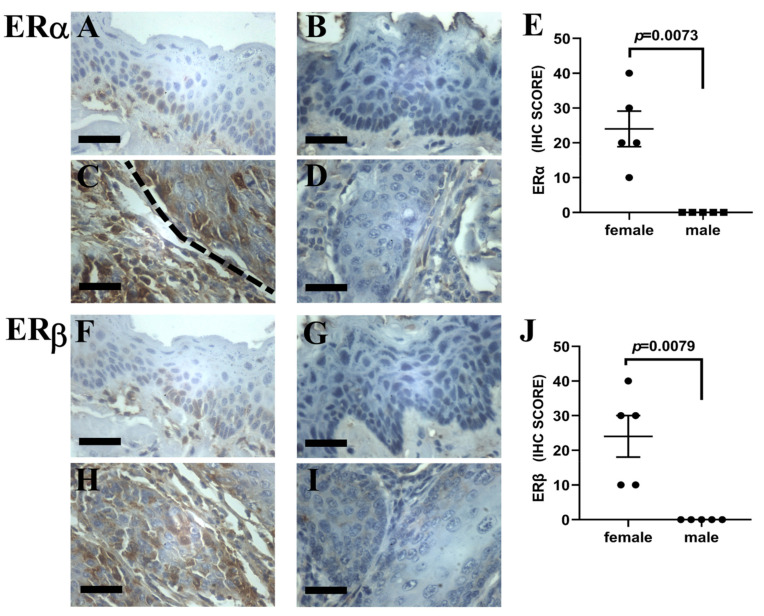
Immunohistochemical detection of estrogen receptors alpha and beta (ERα and ERβ) in intraepithelial and invasive tongue base lesions of female (Panels (**A**,**C**,**F**,**H**)) and male (Panels (**B**,**D**,**G**,**I**)) K14-HPV16 mice, DAB—hematoxylin, 200×. (**A**,**B**) ERα expression in intraepithelial dysplastic lesions. Note mild positivity in basal cells in Panel (**A**) and no staining in Panel (**B**). (**C**,**D**) ERα expression in invasive lesions. Note positivity in tumor cells in Panel (**C**) and no staining in Panel (**D**). Dashed line represents the tumor-stroma interface. (**E**) ERα immunohistochemical scores in intraepithelial lesions from female and male K14-HPV16 animals. (**F**,**G**) ERβ expression in intraepithelial dysplastic lesions. Note mild positivity in basal cells in Panel F and no staining in Panel (**G**). (**H**,**I**) ERβ expression in invasive lesions. Note positivity in tumor cells in Panel (**H**) and no staining in Panel (**I**). (**J**) ERβ immunohistochemical scores in intraepithelial lesions from female and male K14-HPV16 animals. Bar = 50 μm.

We hypothesized that the increased incidence of SCC in female mice could be related to up-regulated signaling via sex hormone receptors leading to increased progression of tongue base intraepithelial lesions to invasive SCC. We started by analyzing the expression of ERα, ERβ and progesterone receptors in the normal (HPV16−) squamocolumnar junction between the circumvallate papilla and von Ebner’s gland, where SCC was hypothesized to arise [9]. The squamocolumnar junction was consistently negative for all three markers (Figure S2). The adjacent oral mucosa of the tongue base was also negative in HPV− mice. K14-HPV16 female mice showed robust ERα and ERβ expression in tongue base intraepithelial and invasive lesions (Figure 3A,C,F,H) while male mice showed minimal ERα and ERβ expression (Figure 3B,D,G,I, Figure S3). ERα and ERβ scores were significantly higher in female versus male tongue base intraepithelial lesions. No statistical analysis was performed for invasive lesions due to the presence of a single SCC studied immunohistochemically in the male group. Immunohistochemical stains for progesterone receptors were negative in the oral lesions of male and female mice (data not shown).

### 3.4. Expression of MMP2 in Tongue Base Lesions

**Figure 4:**
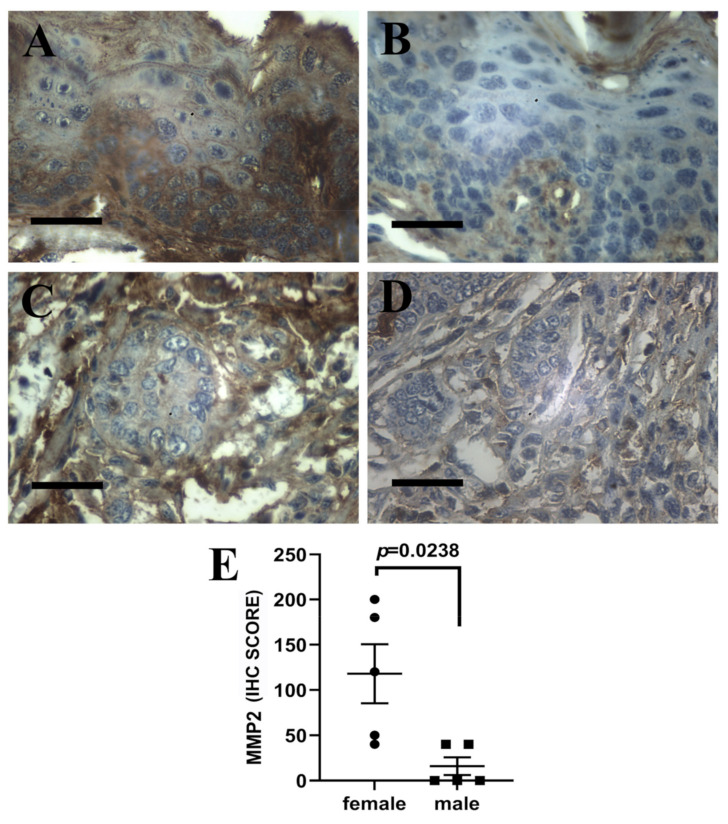
Immunohistochemical detection of metalloproteinase 2 (MMP2) in tongue base intraepithelial and invasive lesions of female (Panels (**A**,**C**)) and male (Panels (**B**,**D**)) K14-HPV16 mice, DAB—hematoxylin, 200×. (**A**,**B**) MMP2 expression in intraepithelial dysplastic lesions. Note marked positivity in epithelial cells in Panel (**A**) and no epithelial staining in Panel (**B**). (**C**,**D**) MMP2 expression in invasive lesions. Note moderate positivity in tumor cells in Panel (**C**) and no staining in Panel (**D**). (**E**)—MMP2 immunohistochemical scores in intraepithelial lesions from female and male animals. Bar = 50 μm.

We next investigated the expression of MMP2, a downstream target of ERα and ERβ [19,20], in the tongue base lesions of male and female mice. We observed that intraepithelial lesions from female animals showed significantly (*p* = 0.0238) increased MMP2 immunoexpression compared with matched male lesions (Figure 4A,B), in line with increased ER expression in females. Invasive lesions from female mice and the single SCC from a male mouse showed moderate to strong MMP2 immunostaining (Figure 4C,D).

### 3.5. Collagen Remodeling in Tongue Base Lesions from Male and Female Mice

**Figure 5:**
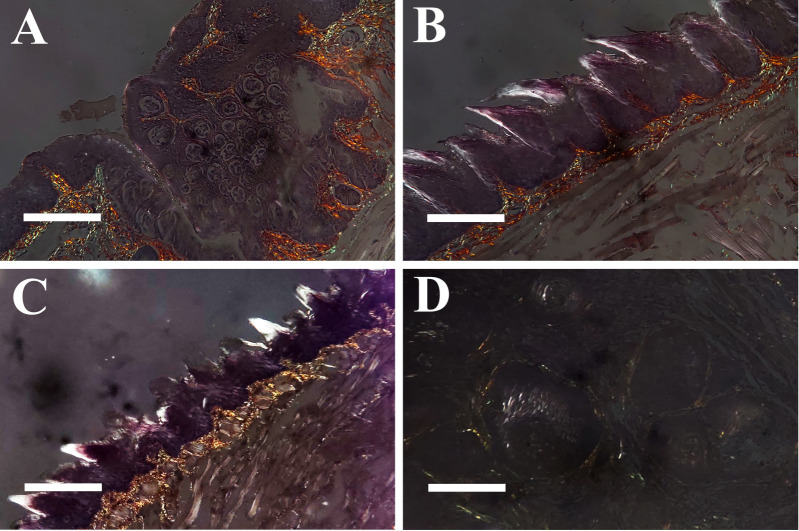
Picrosirius stain for collagen fibres under polarized light microscopy in wild-type and K14-HPV16 transgenic mice, 200×. (**A**) Normal tongue base, circumvallate papilla. Note delicate network of green and orange-refringent fibres supporting the overlying epithelium. (**B**) Normal tongue base. Note delicate network of green and orange-refringent fibres supporting the overlying epithelium. (**C**) Tongue base, high-grade dysplasia. Note replacement of the normal collagen network by thick, condensed, yellow-refringent collagen bundles. (**D**) Tongue base, squamous cell carcinoma. Note the near absence of collagen fibres surrounding tumour cells. Bar = 50 μm.

To confirm whether MMP2 plays an active role in promoting the development of tongue base SCC from intraepithelial lesions in this model, we studied the distribution of its substrate, collagen, using sirius red stains under polarized light microscopy (Figure 5). This approach revealed that the normal lamina propria of the tongue base contained a delicate reticular network of green-refringent collagen fibers spreading from the overlying basement membrane to the underlying skeletal muscle tissue. Thicker and longer orange-refringent collagen fibers were also present (Figure 5A,B). Intraepithelial lesions showed a marked condensation of this network, with loss of the typical reticular pattern, which was replaced by condensed, thickened bundles of yellow-refringent collagen fibers (Figure 5C). In invasive lesions, there was an even deeper remodeling of the lamina propria, with near-complete loss of both types of collagen fibers (Figure 5D).

## 4. Discussion

Mice carrying all or some of the HPV16 early genes have been used to mimic various cancers [21,22,23,24], to study the mechanisms of HPV-induced carcinogenesis [25], test preventive and therapeutic strategies [26,27,28] and decipher the role of HPV co-carcinogens [11,22]. The present study is the first to report the differential incidence of SCC in the tongue of male and female HPV16-transgenic mice. The significantly higher incidence of SCC in female mice was surprising because men are at increased risk of OPSCC compared with women [2,29]. The unusually high number of mice employed in the transgenic groups was necessary to identify malignant lesions in male animals and a smaller scale study would be likely to miss those lesions. Interestingly, the incidence of low- and high-grade intraepithelial lesions was similar in both sexes. This suggested that sex-related factors specifically act to facilitate the acquisition of invasive capabilities by high-grade lesions in female animals rather than inducing a higher number of de novo intraepithelial lesions. As previously reported for female K14HPV16 mice [9], SCC in male animals were concentrated in the tongue base, but intraepithelial lesions arose in multiple locations. These similar observations from male and female mice suggest that intraepithelial lesions arising from the tongue base have a much greater potential to develop into invasive lesions than intraepithelial lesions developing in other sites. Our group recently associated this predisposition with the presence of a squamocolumnar transition zone in the circumvallate papilla in this animal model [9]. These results support the notion that certain anatomic sites have an increased potential for developing malignant lesions, as previously described for squamocolumnar transition zones in the cervix and anus [10]. The HPV 16 E6, E7 and E5 oncogenes are known to drive cell transformation through multiple mechanisms, as recently reviewed [30]. Importantly, these oncogenes were consistently expressed in tongue tissues. It is important to bear in mind that, in this model, gene expression is not regulated by the HPV16 long control region as in HPV-infected patients, but rather by the Krt14 gene promoter, which can influence splicing mechanisms [31]. Mouse models based on the Krt14 gene promoter (K14-HPV16 mice) are well characterized, reliable and are an important for studying HPV16-induced lesions, as previously reviewed [25,32], the role of HPV16 oncogenes [14,15] the influence of co-carcinogens [21,22,23,24] and immune evasion mechanisms [27,33], as well as cancer chemopreventive strategies [26,28,34]. However, the HPV16 gene transcription mechanisms based on the Krt14 gene promoter differ from those observed in cancer patients and this limitation should be kept in mind when interpreting the gene expression results. The HPV16 E6, E7 and E5 gene expression pattern requires further analysis to determine the underlying gene regulation mechanisms in this animal model. Alternative splicing processes generate different mRNAs with alternative transcription ends and may help explaining the relative abundance of E6 transcripts [35,36]. In polycistronic mRNA, the first ORF—in this case, the E6 mRNA in the transgene cassette—can be translated more efficiently when intercistronic distances are short [37]. Early and advanced lesions showed identical gene expression patterns, in line with the sustained activity of the Krt14 gene promoter, previously reported in these lesions [9]. Importantly, female mice showed higher levels of E6 transcripts compared with males, which are likely to contribute to their higher SCC incidence by promoting an invasive phenotype with the ability to breach the basement membrane and invade the adjacent tissues [30,38]. We hypothesized that the higher E6 expression levels and higher SCC incidence were associated with increased ER signaling in females. In fact, ERα is critical for cervical carcinogenesis and Arbeit et al. (1996) observed that the expression of viral oncogenes was dependent on the estrous cycle and that estrogen supplementation stabilized oncogene expression, in this animal model [21]. ERalpha was recently shown to facilitate HPV integration via DNA hypermutation through apolipoprotein B mRNA-editing catalytic polypeptide 3 (APOBEC3), and to correlate with improved overall survival in HPV-positive OPSCC [12]. Jin et al. (1999) showed that indole-3-carbinol, an anti-estrogen compound, could hamper cervical carcinogenesis [26]. Oppositely, PR was found to block cervical carcinogenesis in HPV16-transgenic mice [16]. In line with our hypothesis, female lesions intraepithelial lesions showed increased immuneexpression of estrogen receptors alpha and beta compared with matched male samples. This observation suggests that signaling through estrogen receptors may enhance oropharyngeal carcinogenesis in this model. To further study this hypothesis, we analyzed the expression of MMP2, which is up-regulated by both estrogen receptors alpha and beta [19,20] and promotes the invasion of multiple types of cancer [39]. As observed for estrogen receptors, intraepithelial lesions from female mice showed higher MMP2 levels compared with matched male lesions. These findings suggest that increased MMP2 expression via estrogen receptor signaling may be the mechanism whereby female HPV16-transgenic mice become more susceptible to oropharyngeal carcinogenesis. MMP2 is an important player in HPV+ OPSCC, where it promotes tumor cell migration and invasion [40]. Additionally, the HPV E6 oncoprotein may also up-regulate MMP2 expression via the nuclear factor kappa-light-chain-enhancer of activated B-cells (NFκB), as previously reviewed [41]. Finally, we used histochemical stains and polarized light microscopy to study the distribution of collagen, the substrate of MMP2 enzymatic activity. MMP2 is involved in the cleavage of Types I, II, II and IV collagen, as previously reviewed [42]. The results showed that collagen remodeling takes place in intraepithelial lesions, and SCC shows marked collagenolysis, showing that MMP2 is actively involved in tumor invasion in this model. Overall, the present results show that female K14-HPV16 transgenic mice develop tongue-based SCC at a significantly higher rate than males. This is associated with increased expression of estrogen receptors alpha and beta and MMP2, which plays a significant role in degrading collagen and facilitating tumor invasion. HPV16 E6 transcripts are also elevated in female mice, a phenomenon which has also been associated with estrogen receptor signaling in this animal model [21] and is likely to contribute to MMP2 overexpression and tumor invasion. However, additional studies are required to provide mechanistic evidence to support this hypothesis, such as targeted ablation of ER in tongue tissues, pharmacological ER inhibition or transplantation of female premalignant lesions into male mice. The main limitations of this study are related to the animal model employed, which relies on the permanent expression on the HPV16 oncogenes via the Krt14 gene promoter, rather than transient or inducible oncogene expression controlled by the viral long control region. This is a likely reason why the male: female ratio in this model is opposite to that observed in OPSCC patients and should be kept in mind when interpreting the results. Sexual dimorphism affects the host’s response to many pathogens, including the new coronavirus Sars-Cov-2 [43]. Previous studies using similar models, such as those induced by 4-nitroquinoline-1-oxide (4NQO) in conjunction with HPV16 *E6*, *E7* or both [18,22] did not address this point. Compared with previous studies, the present approach has the advantage of employing the full HPV16 early region and dispensing with chemical carcinogens. This led to a much greater specificity, i.e., tumors were concentrated in the oropharynx, while 4NQO promoted tumor development throughout the oral cavity and oesophagus [18,22]. The sex bias now detected suggests that future translational studies on this model should rely on male mice to mimic more closely the clinical scenario.

## 5. Conclusions

The present study adds to the current understanding of how sexual dimorphism and particularly the expression of sex hormone receptors can modulate the host’s response to HPV in this animal model. However, the much higher OPSCC incidence now observed in female mice is in contrast with observations from OPSCC patients. This was associated with markedly enhanced expression of ER and MMP2 in female samples, suggesting that ER signaling play a major role in OPSCC carcinogenesis in K14-HPV16 mice. Taking this into consideration, male K14-HPV16 mice seem to recapitulate tongue base carcinogenesis more closely than females, and are preferable for translational research on OPSCC.

## Figures and Tables

**Table 1:** Incidence of epithelial lesions in the tongue of male and female mice. Number and percentage (%) of affected mice for each experimental group.

Groups	Normal *n* (%)	Low-Grade Dysplasia *n* (%)	High-Grade Dysplasia *n* (%)	Papilloma *n* (%)	SCC *n* (%)
Female HPV+ (*n* = 82)	0	37 (45.1%)	16 (19.5%)	10 (12.2%)	19 (23.2%)
Female HPV− (*n* = 10)	10 (100%)	0	0	0	0
Male HPV+ (*n* = 67)	3 (4.5%)	30 (44.8%)	19 (28.3%)	12 (17.9%)	3 (4.5%)
Male HPV− (*n* = 10)	10 (100%)	0	0	0	0

**Table 2:** Distribution of histological lesions in the tongue of female and male HPV16-transgenic.

Sex/Anatomical Site	Low-Grade Dysplasia *n*	High-Grade Dysplasia *n*	Papilloma *n*	SCC *n*
**Females** (*n* = 82)				
**Tongue base**	30	23	10	19
**Rostro-dorsal tongue**	33	28	2	1
**Ventral tongue**	10	2	0	1
**Total lesions**	73	53	12	21
**Average lesions/mouse**	**0.89**	**0.64**	**0.15**	**0.26**
**Males** (*n* = 67)				
**Tongue base**	30	19	12	3
**Rostro-dorsal tongue**	30	13	1	0
**Ventral tongue**	8	4	0	0
**Total lesions**	68	36	13	3
**Average lesions/mouse**	**1.01**	**0.54**	**0.19**	**0.04**

## Data Availability

No new data were created or analyzed in this study. Data sharing is not applicable to this article.

## Supplementary Materials

The following are available online at https://www.mdpi.com/article/10.3390/pathogens10101224/s1, Figure S1: normalized relative expression (−ΔCt) of HPV16 *E6*, *E7* and *E5* in female (A) and male (B) tongue base samples, Figure S2: immunohistochemical detection of estrogen receptor alpha (ERα) an beta (ERβ) on the HPV− murine circumvallate papilla, Figure S3: immunohistochemical detection of estrogen receptor alpha (ERα) an beta (ERβ) on male and female HPV^+^ intraepithelial lesions, Table S1: primer sequences and PCR conditions used for genotyping experimental mice., Table S2: RT-PCR primer sequences of for HPV16 E6, E7, E5, and for TBP, HPRT and β2m, Table S3: primary antibodies and dilutions used for immunohistochemical experiments.
