# Supplementary material for: Differential Incidence of Tongue Base Cancer in Male and Female HPV16-Transgenic Mice: Role of Female Sex Hormone Receptors
Source: Pathogens. 2021 Sep 22;10(10):1224. doi: 10.3390/pathogens10101224 (PMC8539196; doi:10.3390/pathogens10101224)
Supplement: Supplementary file 1 [file pathogens-10-01224-s001.zip › Supplementary figures.pptx]

## Slide 1
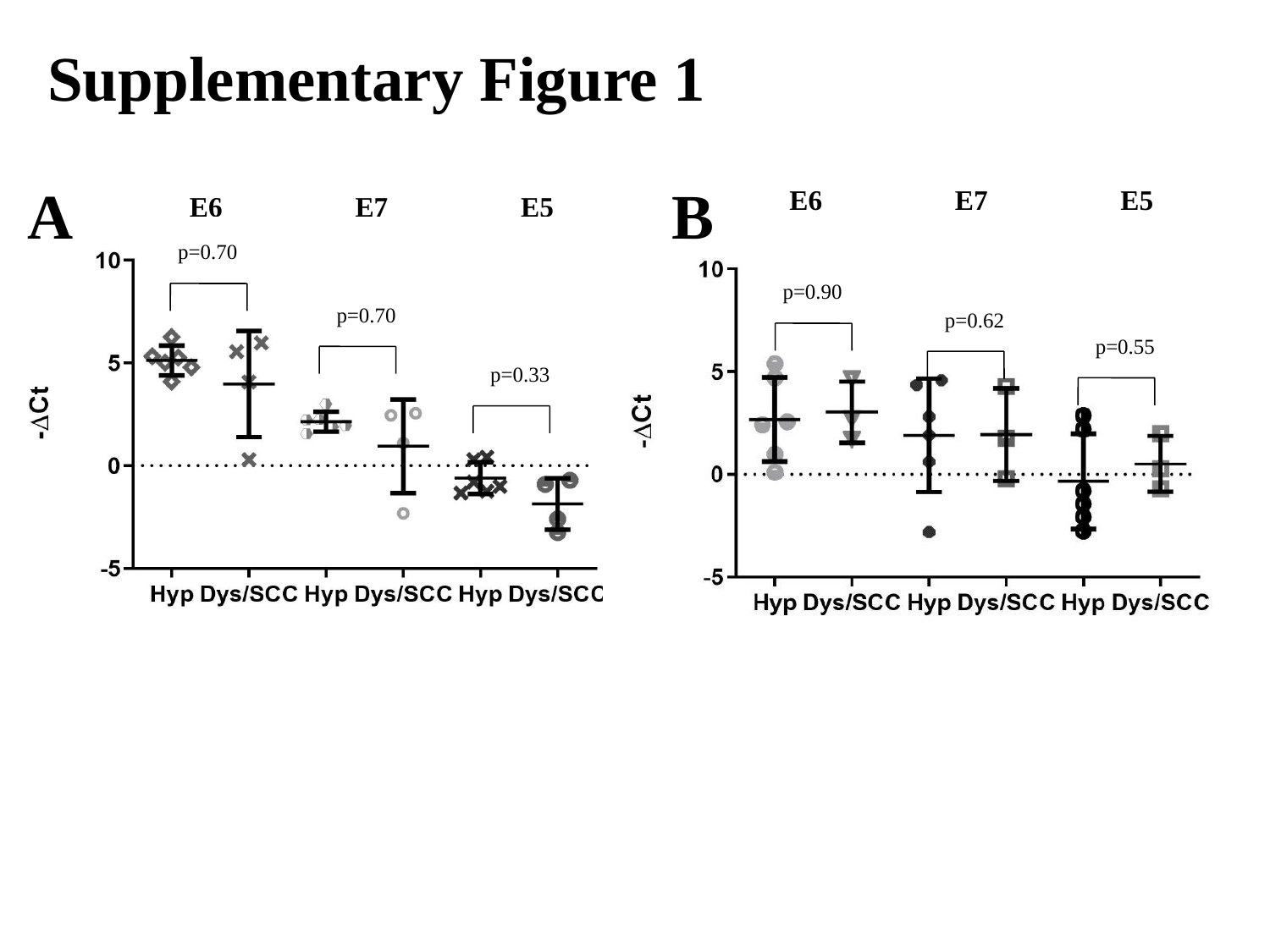

Supplementary Figure 1
A
 E6 E7 E5
 p=0.70
 p=0.70
 p=0.33
B
 E6 E7 E5
 p=0.90
 p=0.62
 p=0.55

## Slide 2
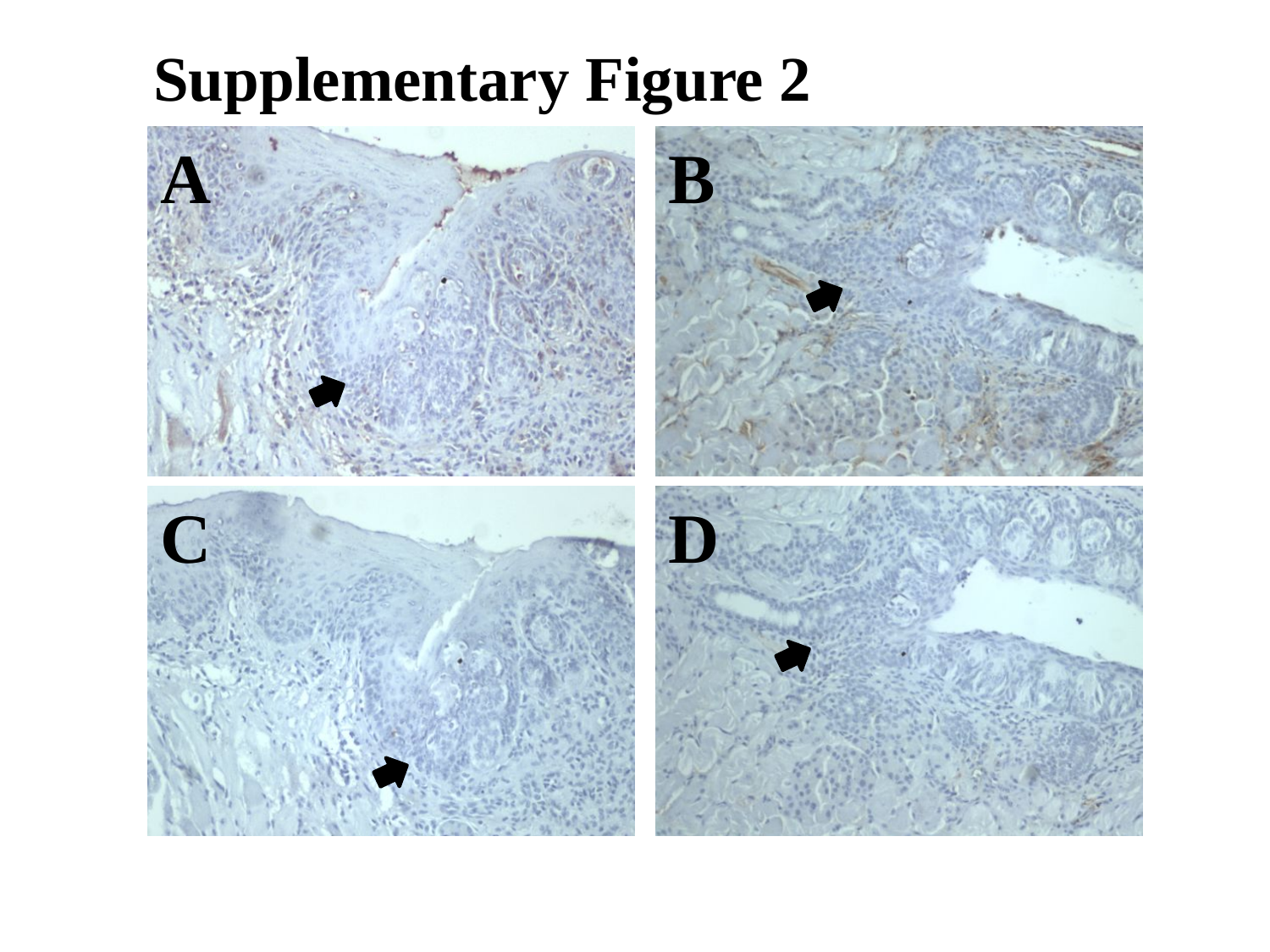

Supplementary Figure 2
A
B
C
D

## Slide 3
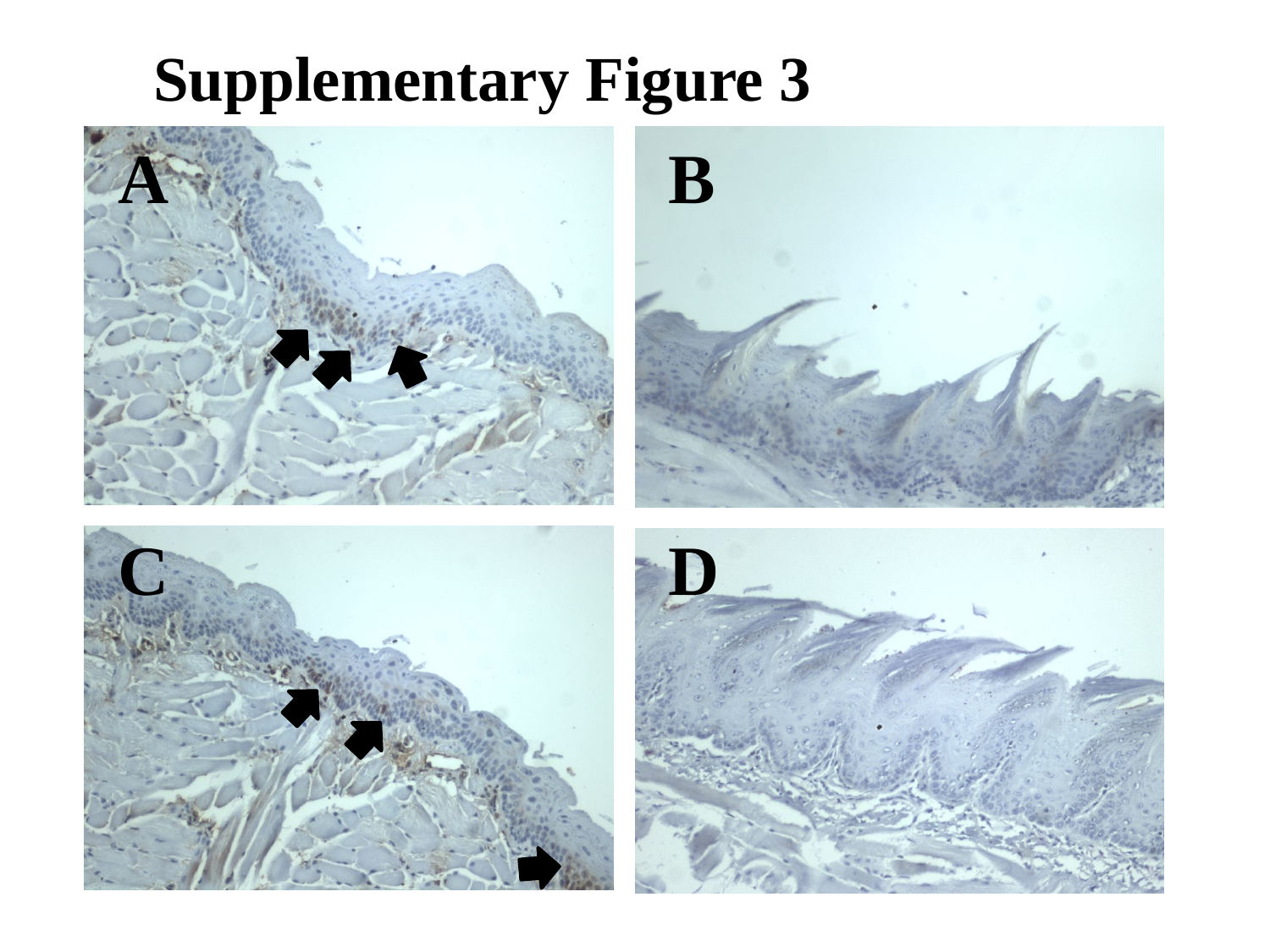

Supplementary Figure 3
A
B
C
D
