# Supplementary material for: Differential Incidence of Tongue Base Cancer in Male and Female HPV16-Transgenic Mice: Role of Female Sex Hormone Receptors
Source: Pathogens. 2021 Sep 22;10(10):1224. doi: 10.3390/pathogens10101224 (PMC8539196; doi:10.3390/pathogens10101224)
Supplement: Supplementary file 1 [file pathogens-10-01224-s001.zip › Supplementary materials.pdf]

Supplementary Table 1. Primer sequences and PCR conditions used for genotyping experimental mice.

Gene	Primer Sequence (5'->3')	Amplicon size (bp)
E7	F: GGAGGAGGATGAAATAGATGG<br>R: GCCCATTAAGAGGTCTTCCAA	157
Hbb	F: CCAATCTGCTCACACAGGATAGAGAGGGCAGG<br>R: CCTTGAGGCTGTCCAAGTGATTCAGGCCATCG	494

F - forward, R - reverse. Amplification conditions: DNA denaturation at 94°C for three minutes, 35 cycles at 94°C for 30 seconds, 60°C for 45 seconds, 72°C for 90 seconds and a final extension step at 72°C for ten minutes. Amplification of DNA fragments with the expected band size was confirmed by electrophoresis in a 3% agarose gel.

Supplementary Table 2. RT-PCR Primer sequences of for HPV16 E6, E7, E5, and for TBP, HPRT and β2m. F - forward, R - reverse.

Gene	Primer sequence (5'->3')	Amplicon size (bp)
E6	F: GAGCGACCCAGAAAGTTACCAC<br>R: ACCTCACGTCGCAGTAACTGTTG	1**07**
E7	F: ACCGGACAGAGCCCATTACAA<br>R: GTGCCCATTAACAGGTCTTCC	120
E5	F: CTTTGCTTTTGTGTGCTTTTGTGTG<br>R: AAAGCGTGCATGTGTATGTATTAAA	192
TBP	F: CAAACCCAGAATTGTTCTCCTT<br>R: ATGTGGTCTTCCTGAATCCCT	131
HPRT	F:TGAAGAGCTACTGTAATGATCAGTCAAC<br>R: AGCAAGCTTGCAACCTTAACCA	187
β2m	F: GGTCTTTCTGGTGCTTGTCTCA<br>R: GTTCGGCTTCCCATTCTCC	103

F - forward, R - reverse.

Supplementary Table 3. Primary antibodies and dilutions used for immunohistochemical experiments.

Target protein	Supplier, clone	Dilution
**ERα**	Santa Cruz Biotech, F10	1:200
**ERβ**	Santa Cruz Biotech, B-1	1:200
**PR**	Santa Cruz Biotech, AB-52	1:200
**MMP2**	Santa Cruz Biotech, 8B4	1:100

ERα- Estrogen receptor alpha, ERβ - Estrogen reeptor beta, PR- Progesterone receptor, MMP2- Matrix metalloproteinase 2.
